# Genotype IX Newcastle disease virus isolated from wild birds is attenuated by hemagglutinin-neuraminidase mutation

**DOI:** 10.1128/jvi.00071-26

**Published:** 2026-05-20

**Authors:** Kejia Lu, Lina Tong, Prince-Theodore Daguia-Wenam, Zhengwu Chang, Sa Xiao, Zengqi Yang, Haijin Liu

**Affiliations:** 1 College of Veterinary Medicine, Northwest A&F University 12469, Shaanxi Yangling, China; 2 College of Agriculture and Animal Husbandry, Qinghai University 207475, Xining, China; 3 Engineering Research Center of Efficient New Vaccines for Animals, Ministry of Education, Yangling, China; 4 Key Laboratory of Ruminant Disease Prevention and Control (West), Ministry of Agriculture and Rural Affairs, Yangling, China; Emory University School of Medicine, Atlanta, Georgia, USA

**Keywords:** Newcastle disease virus, wild birds, transmission, HN protein, virulence

## Abstract

Newcastle disease virus (NDV) infects a wide range of hosts, including poultry and wild birds. However, most research has focused on poultry-derived strains, with limited studies on the biological characteristics of NDV isolated from wild birds. This study investigated genotype IX NDV strains from wild birds. Genetic evolution and viral ecology analyses suggest possible poultry-to-wild-bird transmission of this genotype. Most of the genotype IX NDV strains, isolated from asymptomatic wild birds, were highly pathogenic in chickens, but one strain isolated from spotted dove exhibited attenuated virulence. This dove-derived strain showed the highest homology with a virulent strain from Eurasian blackbird, differing by only 12 amino acids across proteins. Using reverse genetics, we identified the viral HN protein as a critical determinant of virulence in the dove strain. The coexistence of F110 and G116 residues in the HN protein was associated with reduced HN cell-surface abundance and downstream decreases in viral membrane fusion activity, replication capacity, and tissue tropism. Computational modeling further suggests that the F110/G116 combination may be linked to a local α-helix within the HN head-stalk linker region, providing a plausible structural context for these effects. Crucially, this dove strain’s fusion protein retained the furin-cleavable, virulent-type “¹¹²RRQRRF¹¹⁷” cleavage site. Immunizing chickens with this strain induced high antibody titers within 1 week, and titers persisted at high levels for 13 weeks and provided complete protection against virulent challenge at both early and late post-immunization time points. Our findings uncover an HN-linked attenuation mechanism centered on residues 110 and 116, primarily through their effects on HN cell-surface abundance and downstream fusion activity, offering new insights into the development of immunogenic NDV vaccines with reduced virulence.

**IMPORTANCE:**

Wild birds, as natural reservoirs of Newcastle disease virus (NDV), harbor a diverse range of viral strains. However, research on these strains remains limited. Here, we show that genotype IX NDV detected in wild birds most likely reflects possible poultry-to-wild bird transmission rather than long-term endemic circulation in wild populations. Using isolates derived from wild birds, we report a novel attenuation mechanism involving structural modification of the HN protein’s head-neck linker region. Therefore, research on wild bird-derived isolates not only provides insights into the epidemiological dynamics of NDV transmission but also facilitates the identification of novel attenuated strains, which could aid in the development of high-efficacy NDV vaccines and NDV-based viral vectors.

## INTRODUCTION

Newcastle disease virus (NDV), a member of the *Paramyxoviridae* family, has a negative-sense, single-stranded RNA genome with 3′Leader-NP-P-M-F-HN-L-Trailer′5 structure, encoding six structural proteins and two nonstructural proteins (1). Birds, including both domestic poultry and wild birds, serve as the primary hosts of NDV (2). Phylogenetic analysis of the fusion (F) gene has revealed that NDV strains are classified into two classes: class I, which contains one genotype, and class II, which encompasses 21 genotypes (3). Although certain genotypes, such as genotype VI, show a host preference for the specific bird family (e.g., Columbidae), most NDV genotypes are widely distributed among poultry and wild birds, suggesting that the virus can readily spread between different bird species (4).

Class II genotype IX NDV, represented by the F48E9 strain, was first isolated in China in 1946 (5). Since then, genotype IX NDV has been rarely detected in poultry or wild birds and has only been reported in China (6–8). Like other RNA viruses, NDV’s RNA-dependent RNA polymerase (L) lacks proofreading activity, resulting in a high mutation rate and rapid genetic evolution (9, 10). However, in contrast to other genotypes, the evolution rate of genotype IX NDV is exceptionally slow, with a remarkable 99% genetic homology between the oldest and most recent isolates. This genetic stability is more compatible with repeated introductions into, rather than long-term endemic cycling among, avian populations (11). Most genotype IX NDV strains are isolated from sick or deceased poultry, and even most wild bird-origin strains are highly pathogenic to chickens. In contrast, virus-positive wild birds frequently remain asymptomatic despite infection, suggesting disease tolerance and/or reduced pathogenicity rather than resistance to infection by itself (6, 8). Interestingly, some genotype IX NDV isolates from wild birds show low pathogenicity in chickens, raising the possibility that transient replication in wild birds might select for attenuating mutations; however, conclusive experimental evidence is still lacking (7).

NDV strains are classified into lentogenic, mesogenic, or velogenic types based on their pathogenicity in chickens. The virulence of NDV is influenced by a variety of factors, with the F protein playing a central role (12). The F protein must be cleaved at a specific site to release its peptide, which then inserts into the host cell membrane. This cleavage and subsequent peptide insertion are essential for viral fusion with the host cell membrane (13, 14). Additionally, the hemagglutinin-neuraminidase (HN) protein is crucial for recognizing viral receptors and regulating the timing of F protein peptide release, further contributing to NDV virulence. Therefore, the viral fusion capability is generally positively correlated with NDV virulence (15). However, for some strains, even if the viral particle or the F and HN proteins induce clear syncytium formation in cells, they may not cause disease or death in chickens (7). This suggests that other genomic regions, such as the V gene, L gene, and even noncoding regions, also influence NDV virulence (16–20). Despite the identification of several virulence factors, the complete molecular basis of NDV virulence remains unclear.

In this study, genotype IX NDV strains isolated from wild birds were used to explore viral attenuation and to identify new determinants of NDV virulence. Two genotype IX NDV strains, isolated from wild birds, exhibited high genomic homology but divergent pathogenicity. The contribution of various amino acid variations between these strains to NDV tissue tropism, virulence, and immunogenicity was examined.

## RESULTS

### Genotype IX NDV in wild birds: evolutionary context and virulence

**Fig 1:**
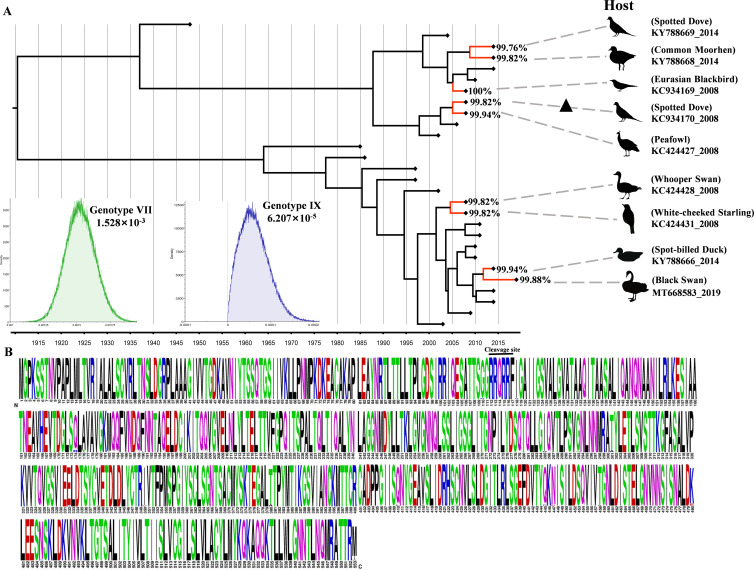
Genotype IX NDV in wild birds: evolutionary context and virulence. (**A**) Maximum clade credibility (MCC) tree of Genotype IX. The MCC tree was constructed based on the F gene sequences of all genotype IX NDV strains (Table S1) using BEAST v1.10.4 under the relaxed clock model. The posterior density of the clock rate (substitutions/site/year) for genotype VII (Table S2) and genotype IX was extracted from the log files using Tracer v1.71 and incorporated into the figure. Red branches represent genotype IX NDV isolates from wild birds. The data show the genetic similarity between F48E9 (the earliest isolated genotype IX virus) and the wild bird viruses. (**B**) F protein alignment. The amino acid sequence alignment of the genotype IX NDV F protein was performed using WebLogo 3, with the F protein cleavage site indicated.

The information on all genotype IX NDV strains was compiled from references and GenBank. As shown in Table S1, this genotype was found in both poultry and wild birds. The maximum clade credibility (MCC) tree analysis revealed that early genotype IX NDV strains were isolated from poultry, whereas viruses derived from wild birds were first recorded in 2008 (Fig. 1A). The homology between F48E9 (the earliest isolated genotype IX virus) and wild bird strains exceeded 99.5% (Fig. 1A). Analysis of the F protein amino acids showed that the F protein was highly conserved across all genotype IX NDV strains (Fig. 1B). Furthermore, the evolutionary rate was estimated, revealing that the genotype IX NDV evolved at a rate of 6.207 × 10⁻⁵ substitutions/site/year (95% HPD: 3.05 × 10⁻⁶−1.246 × 10⁻⁴), which is much slower than that of other RNA viruses, including other NDV strains such as genotype VII (1.528 × 10⁻³; 95% HPD: 1.325 × 10⁻³−2.061×10⁻³) (Fig. 1A). These results are consistent with repeated inter-host introductions between domestic poultry and wild birds, rather than sustained endemic circulation within wild bird populations. Interestingly, while the F protein cleavage site of all genotype IX NDVs was ^112^RRQRRF^117^ (Fig. 1B), which primarily determines NDV virulence, wild birds infected with genotype IX NDV remained asymptomatic. Moreover, some wild bird-derived genotype IX strains exhibited mesogenic or lentogenic pathogenicity in chickens (Table S1). These findings indicate that genotype IX NDV can potentially spread into wild bird populations and, in some cases, exhibit reduced virulence in chickens.

### Two highly homologous wild bird-derived genotype IX NDV strains exhibit contrasting pathogenicity

To investigate the factors underlying the attenuation of genotype IX NDV upon transmission to wild birds, two NDV strains were selected: SpottedDove/China/08 (Dove), isolated from a wild spotted dove in 2008, and Blackbird/China/08 (Blackbird), isolated from a wild Eurasian blackbird in 2008 (Table S1). Following multiple rounds of plaque purification, the complete genomes of both strains were determined by next-generation sequencing. Comparative genomic analysis revealed that the two viruses were highly homologous, differing by only 21 nucleotides across the 15,192 bp genome, resulting in 12 amino acid differences across all proteins (Table 1). Specifically, there were 2, 7, and 3 amino acid differences in the P, HN, and L proteins, respectively. Although the F genes of both strains contained three variants, the F protein amino acids were identical (Table 1).

**TABLE 1:** Differences of nucleotides and amino acids between NDV Dove and Blackbird strains

Nucleotide position	Virus		Protein	Amino acid position	Virus	
	Dove	Blackbird			Dove	Blackbird
937	G	A	NP	272	K	K
2122	C	T	P	77	S	F
2205	A	G	P	105	S	G
5398	A	G	F	283	L	L
5758	T	C	F	403	D	D
5872	T	C	F	441	D	D
6577	G	A	HN	54	G	S
6745	T	C	HN	110	F	L
6763	G	A	HN	116	G	R
6856	A	G	HN	147	N	D
7344	T	C	HN	309	D	D
7630	A	C	HN	405	I	L
7823	C	T	HN	469	A	V
7982	C	T	HN	522	T	I
9082	G	A	L	232	E	E
9142	T	C	L	252	S	S
9259	G	A	L	291	G	G
10883	C	T	L	833	L	L
11613	A	G	L	1076	K	R
13570	C	A	L	1728	D	E
14013	T	C	L	1876	L	S

Furthermore, the diversity analysis of these 12 amino acid differences was compared with that of other strains. The amino acids were classified into two categories—conservative and nonconservative sites. The conservative group included amino acids at positions 110, 116, 405, and 469 of the HN protein and at positions 1076 and 1876 of the L protein (Fig. S1). While the sequences of most of these conservative sites were identical to those of the Blackbird strain, two sites, G116 of HN and K1076 of L protein, were identical to the Dove strain (Fig. S1).

**Fig 2:**
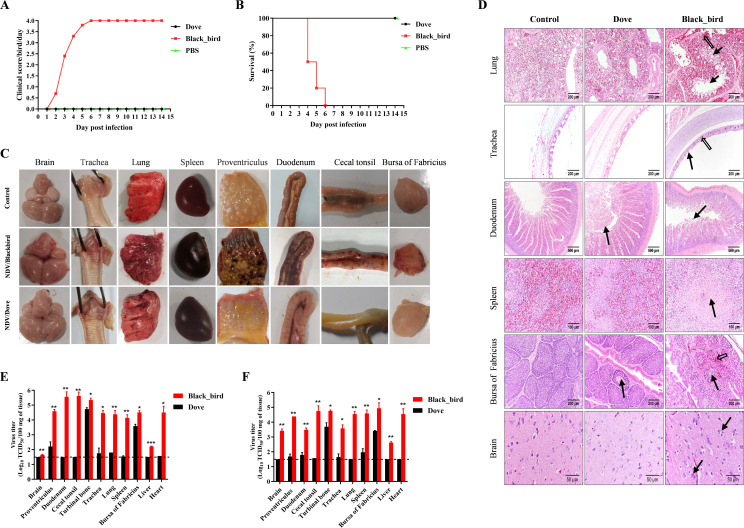
Two highly homologous wild bird-derived genotype IX NDV strains exhibit contrasting pathogenicity. (**A**) Clinical signs of chickens infected with the Blackbird or Dove Strain. Clinical symptoms in 3-week-old chickens were recorded daily following infection with either the Blackbird or Dove strain. (**B**) Survival rate of infected chickens. The daily mortality rate was recorded for 3-week-old chickens infected with either strain. (**C**) Organ pathological changes in Chickens. At 5 days dpi with either the Blackbird or Dove strain, various chicken organs were collected and examined for pathological changes. (**D**) Histopathological changes in chickens. Hematoxylin and eosin (H&E)-stained sections of the previously collected organs were examined under a microscope, with histopathological alterations indicated by arrows. Open arrows: pulmonary parenchymal hemorrhage; vascular congestion in the trachea; bursal follicular atrophy with hemorrhage and widened interfollicular spaces. Black arrows: alveolar capillary congestion; tracheal mucosal epithelial necrosis with epithelial sloughing; duodenal villous epithelial sloughing; lymphoid necrosis/apoptosis with a starry-sky appearance in the splenic periarteriolar lymphoid sheaths (PALS) and germinal centers; starry-sky appearance of bursal lymphoid follicles; neuronal degeneration with diffuse microglial proliferation in the brain. (**E and F**) Viral tissue tropism. Three-week-old chickens were infected with both strains, and organs were collected at 3 dpi (**E**) and 5 dpi (**F**) to titrate the virus (*n* = 3).*P* values were calculated using Student’s *t*-test. An asterisk indicates a comparison with the indicated control. *P < 0.05; **P < 0.01; ***P < 0.001; ns: not significant.

The pathogenicity of the two strains was evaluated using the intracerebral pathogenicity index (ICPI) and experimental infection of 3-week-old chickens. The ICPI values for the purified Dove and Blackbird strains were 0.93 and 1.65, respectively (Table 2). Following infection, chickens inoculated with the Blackbird strain developed severe clinical signs by 3 days post-infection (dpi), including lethargy, ruffled feathers, dyspnea, and anorexia (Fig. 2A). Mortality was first observed at 4 dpi, and all infected chickens succumbed to infection by the end of the observation period (Fig. 2B). In contrast, chickens infected with the Dove strain displayed no clinical signs, and all survived until the 14th dpi (Fig. 2A and B).

**TABLE 2:** Pathogenicity of original, chimeric, and mutated NDV strains^*a*^

Virus	ICPI value	Survival rate^*b*^
Dove	0.93	100%
Blackbird	1.65	0%
rDove	0.95	100%
rBlackbird	1.64	0%
rDove-BP	1.07	100%
rDove-BHN	1.53**	30%
rDove-BL	1.11	90%
rDove-BPL	1.34	–**^*c*^**
rBlackbird-DP	1.60	0%
rBlackbird-DHN	1.25*	60%
rBlackbird-DL	1.59	0%
rBlackbird-DPL	1.54	–
rDove-HN_G54S_	0.98	–
rDove-HN_F110L_	1.25**	70%
rDove-HN_G116R_	1.39**	60%
rDove-HN_N147D_	1.01	–
rDove-HN_I405L_	0.93	–
rDove-HN_A469V_	1.09*	100%
rDove-HN_T522I_	1.18**	100%
rDove-HN_F110L/G116R_	1.48**	40%
rBlackbird-HN_S54G_	1.61	–
rBlackbird-HN_L110F_	1.49*	0%
rBlackbird-HN_R116G_	1.46*	0%
rBlackbird-HN_D147N_	1.60	–
rBlackbird-HN_L405I_	1.63	–
rBlackbird-HN_V469A_	1.56	0%
rBlackbird-HN_I522T_	1.57	0%
rBlackbird-HN_L110F/R116G_	1.29**	60%
rC22	1.78	–
rC22-HN_L110F/G116_	1.10**	100%
rC22-HN_L110/G116R_	1.34	–
rC22-HN_L110F/G116R_	1.30	–

^*a*^ *P < 0.05; **P < 0.01.

^*b*^ Survival rate was tested in 3-week-old chicken via intraocular-nasal root.

^*c*^ –, survival rate was not tested.

Consistent with these clinical outcomes, necropsy and histopathological analyses revealed striking differences in tissue damage between the two strains. Blackbird-infected chickens exhibited extensive hemorrhaging in the throat, proventriculus, intestine, and cecal tonsils, as well as lung necrosis, splenic enlargement, and bursal atrophy (Fig. 2C). Histological examination further revealed bronchiectasis and hemorrhage in the lungs, epithelial cell shedding and hemorrhage in the trachea, villous atrophy in the duodenum, lymphocyte necrosis in the spleen and bursa of Fabricius, and neuronophagia in the brain (Fig. 2D). In contrast, chickens infected with the Dove strain exhibited only mild pathological changes, limited primarily to slight lesions in the duodenum and bursa of Fabricius (Fig. 2C and D).

Viral replication and tissue distribution further distinguished the two strains. At 3 dpi, the Blackbird strain was isolated from multiple organs at high titers, whereas the Dove strain was restricted to a few organs. The titer of the Dove strain was 10^4.5^ TCID_50_/100 mg and 10^3.5^ TCID_50_/100 mg in the turbinal bone and bursa of Fabricius, respectively, which were still lower than those of the Blackbird strain. The proventriculus and trachea contained approximately 10^4^ TCID_50_/100 mg of Blackbird virus, which was 100 times higher than the corresponding titer of the Dove strain (Fig. 2E and Table 3). By 5 dpi, the proventriculus and trachea still harbored the Dove strain at low titers, and although the duodenum and spleen became positive for the Dove virus, the titers were 100 times lower than those of the Blackbird strain (Fig. 2F and Table 3).

**TABLE 3:** Titer of original, chimeric, and mutated NDV strains in tissues of chicken^*a*^

	Brain	Proventriculus	Duodenum	Cecal tonsil	Turbinal bone	Trachea	Lung	Spleen	Bursa of Fabricius	Liver	Heart
Dove	−	+	+	−	+++	−	−	+	+++	−	−
Blackbird	+++	+++	+++	+++	+++	+++	+++	+++	+++	++	+++
rDove	−	+	+	−	+++	+	−	+	+++	Δ	Δ
rBlackbird	+++	+++	+++	+++	+++	+++	+++	+++	+++	Δ	Δ
rDove-BP	−	−	+	−	+++	−	−	+	+++	Δ	Δ
rDove-BHN	+++	+++	+++	+++	+++	+++	+++	+++	+++	Δ	Δ
rDove-BL	−	−	+	−	+++	−	−	+	+++	Δ	Δ
rBlackbird-DP	+++	+++	+++	+++	+++	+++	+++	+++	+++	Δ	Δ
rBlackbird-DL	+++	+++	+++	+++	+++	+++	+++	+++	+++	Δ	Δ
rBlackbird-DHN	+	++	+	++	+++	++	+	++	+++	Δ	Δ
rDove-HN_F110L_	++	Δ	++	Δ	Δ	Δ	++	Δ	+++	Δ	Δ
rDove-HN_G116R_	++	Δ	+++	Δ	Δ	Δ	++	Δ	+++	Δ	Δ
rDove-HN_A469V_	−	Δ	+	Δ	Δ	Δ	+	Δ	+++	Δ	Δ
rDove-HN_T522I_	−	Δ	+	Δ	Δ	Δ	+	Δ	+++	Δ	Δ
rDove-HN_F110L/G116R_	++	Δ	+++	Δ	Δ	Δ	+++	Δ	+++	Δ	Δ
rBlackbird-HN_L110F_	+++	Δ	+++	Δ	Δ	Δ	+++	Δ	+++	Δ	Δ
rBlackbird-HN_R116G_	++	Δ	+++	Δ	Δ	Δ	++	Δ	+++	Δ	Δ
rBlackbird-HN_V469A_	+++	Δ	+++	Δ	Δ	Δ	+++	Δ	+++	Δ	Δ
rBlackbird-HN_I522T_	+++	Δ	+++	Δ	Δ	Δ	+++	Δ	+++	Δ	Δ
rBlackbird-HN_L110F/R116G_	+	Δ	++	Δ	Δ	Δ	++	Δ	+++	Δ	Δ

^*a*^ −, virus could not be detected; +, virus titer ≤10^2^ TCID_50_ in 100 mg tissue; ++, virus titer ≤10^3^ and >10^2^ TCID_50_ in 100 mg tissue; +++, virus titer >10^3^ TCID_50_ in 100 mg tissue; Δ, virus titration was not performed.

These findings suggest that the Dove strain represents a distinct genotype IX NDV transition into wild birds, characterized by reduced virulence. The lower virulence and limited tissue tropism of the Dove strain may be attributed to differences in the viral proteins compared to the Blackbird strain.

### The HN protein modulates the virulence and tissue tropism of NDV isolated from the spotted dove

To identify the proteins responsible for the virulence differences between the Dove and Blackbird strains, we generated reverse genetics constructs for both viruses. Rescued viruses were confirmed by sequencing and exhibited growth characteristics, pathogenicity, and cytopathic effects comparable to those of their parental strains (Tables 2 and 3; Fig. S2). Specifically, rBlackbird caused 100% mortality in 3-week-old chickens, whereas rDove-infected chickens remained asymptomatic, consistent with the phenotypes of the corresponding parental viruses (Fig. S2E).

**Fig 3:**
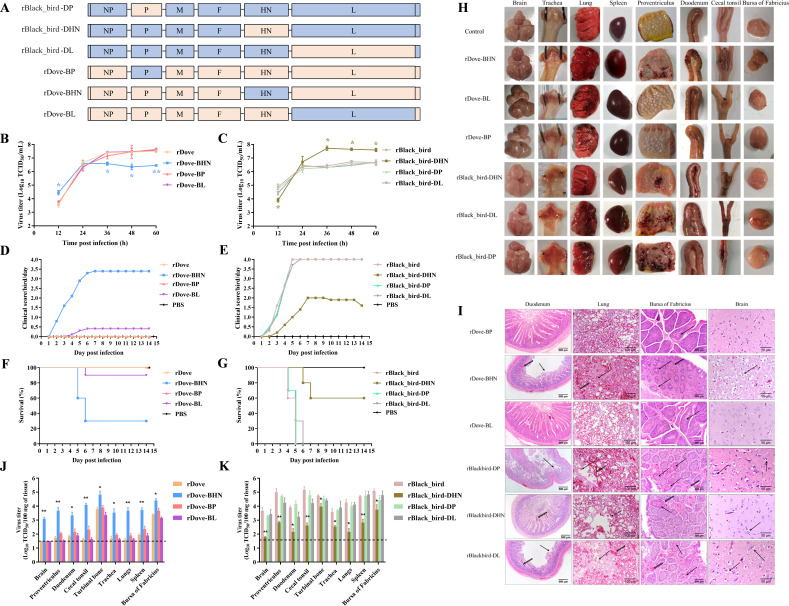
The HN protein modulates the virulence and tissue tropism of NDV isolated from the spotted dove. (****A****) Schematic of recombinant NDV chimeras generated by exchanging the P, HN, or L gene between the Blackbird and Dove strains. (**B and C**) Viral replication *in vitro*. The replication abilities of the chimeric strains were compared to those of the rDove (**B**) and rBlackbird (**C**) strains in DF-1 cells (*n* = 3). (**D and E**) Clinical signs in chickens. Clinical symptoms of chickens were scored daily following infection and compared between the original and chimeric strains. (**F and G**) Survival rate of chickens. The daily survival rate of chickens was recorded following infection and compared between the original and chimeric strains up to 14 dpi. (**H**) Pathological changes in chicken tissues. At 5 dpi, various chicken tissues were collected and examined for pathological alterations. (**I**) Histopathological changes in chicken tissues. H&E-stained tissue sections were prepared from the collected organs and examined under a microscope, with histopathological changes indicated by arrows. Open arrows: duodenal hemorrhage; pulmonary parenchymal hemorrhage; hemorrhage in bursal lymphoid follicles. Black arrows: duodenal villous epithelial sloughing; alveolar congestion; starry-sky appearance of bursal lymphoid follicles; neuronal degeneration with diffuse microglial proliferation in the brain. (**J and K**) Viral tissue tropism. At 5 dpi, different chicken organs were collected and titrated for viral load (*n* = 3). *P* values were calculated using Student’s *t*-test. An asterisk indicates a comparison with the indicated control. *P < 0.05; **P < 0.01; ***P < 0.001; ns: not significant.

Since the differential amino acids were confined to the P, HN, and L proteins of the Dove and Blackbird strains, the three genes were individually exchanged to generate six recombinant strains (Fig. 3A). Replacement of the Dove HN gene with that of Blackbird significantly enhanced viral replication during the early stage of infection, whereas the reciprocal substitution in the Blackbird background markedly reduced early replication. In contrast, exchange of the P or L genes did not significantly affect viral growth kinetics *in vitro* (Fig. 3B and C). Consistent with these findings, rDove-BHN formed larger syncytia, whereas rBlackbird-DHN exhibited reduced cytopathic effects relative to their respective parental strains; no obvious differences were observed for the P- or L-swapped viruses (Fig. S3). ICPI assays showed that swapping in Blackbird HN increased virulence, the reciprocal Dove HN swap reduced it, and P or L swaps had no effect (Table 2).

To evaluate *in vivo* pathogenicity, 3-week-old chickens were infected with the recombinant viruses. rDove-BHN induced severe clinical signs and caused up to 70% mortality, whereas rBlackbird-DHN exhibited markedly reduced virulence compared with rBlackbird, resulting in 40% mortality (Fig. 3D through G). Necropsy revealed prominent hemorrhaging in the trachea, proventriculus, and duodenum, as well as atrophy of the bursa of Fabricius in chickens infected with rDove-BHN. These pathological changes were absent in chickens infected with rDove, rDove-BP, or rDove-BL (Fig. 3H). Additionally, rBlackbird-DHN induced less severe pathological changes in chicken tissues compared to rBlackbird, rBlackbird-DL, or rBlackbird-DP (Fig. 3H). Histopathological examination revealed similar trends, with rDove-BHN causing villous shedding in the duodenum, hemorrhaging in the lung and bursa of Fabricius, and neuronophagia in the brain. In contrast, rDove, rDove-BP, and rDove-BL strains did not cause significant histopathological changes in these tissues (Fig. 3I). Although similar histopathological changes were observed in chickens infected with rBlackbird-DHN, they were less severe compared to rBlackbird, rBlackbird-DL, or rBlackbird-DP (Fig. 3I). Analysis of viral tissue distribution revealed that substitution of the HN protein markedly altered tissue tropism (Table 3). rDove-BHN was detected in multiple organs, including the turbinal bone, bursa of Fabricius, brain, trachea, lung, spleen, and duodenum, with high viral titers in these tissues (Fig. 3J). Although rBlackbird-DHN was detected in similar organs, its viral titers, especially in the brain, were much lower than those of rBlackbird (Fig. 3K).

In contrast, exchange of the P protein did not alter virulence, tissue tropism, or pathological outcomes in either viral background as rDove-BP and rBlackbird-DP exhibited phenotypes indistinguishable from those of their parental strains (Fig. 3D through K). Substitution of the L protein produced only modest changes in ICPI and clinical signs in the Dove background and did not significantly affect viral replication, tissue tropism, or histopathology. Consistently, rBlackbird-DL displayed virulence and tissue distribution similar to those of rBlackbird (Tables 2 and 3; Fig. 3D through K). Although exchange of both the P and L genes resulted in reciprocal changes in ICPI values, these effects were markedly weaker than those observed following HN substitution (Table 2).

Collectively, these results suggest that the HN protein is a key determinant influencing tissue tropism and virulence of genotype IX NDV strains isolated from wild birds.

### Amino acids 110 and 116 of the HN protein determine virulence and tropism of NDV Spotted dove strain

**Fig 4:**
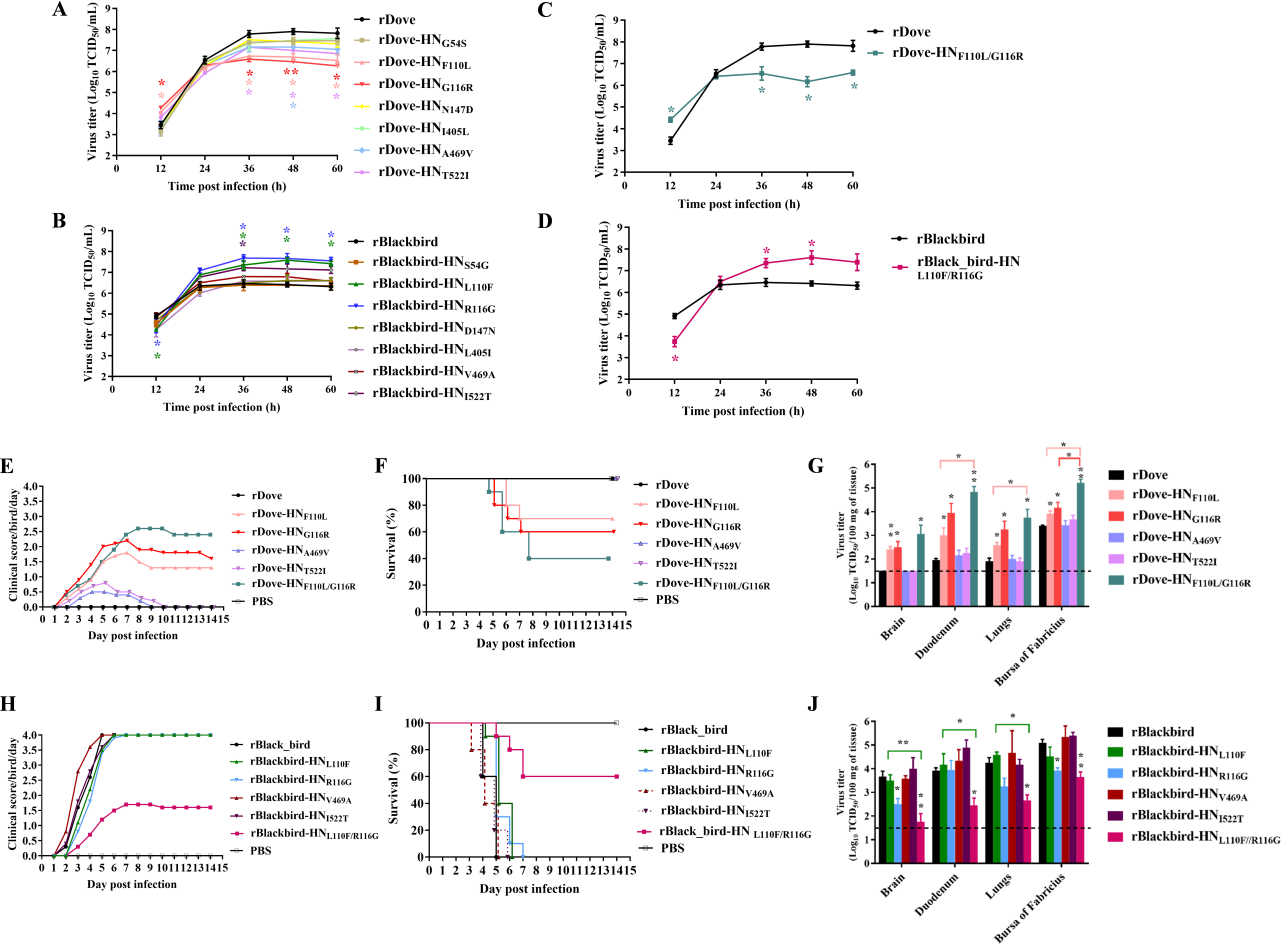
The 110th and 116th amino acids of the HN protein determine virulence and tropism of NDV Spotted dove strain. (**A–D**) Viral replication *in vitro*. The replication abilities of the mutated and original strains were compared in DF-1 cells. Data are expressed as means ± standard deviation (SD) from three independent experiments. (**E and H**) Clinical signs in chickens caused by the mutated strains. In 3-week-old chickens, clinical symptoms were scored daily and compared between the mutated and original strains. (**F and I**) Survival rate of chickens infected with different mutated strains. (**G and J**) Viral load in tissues of infected chickens. At 5 dpi, organs from three infected chickens were collected and titrated for viral load, comparing the mutated and original strains. *P* values were calculated using Student’s *t*-test. An asterisk indicates a comparison with the indicated control. *P < 0.05; **P < 0.01; ***P < 0.001; ns: not significant.

The HN protein exhibited seven amino acid differences between the two strains (Table 1). To identify the amino acids associated with the virulence of the Dove strain, we generated 14 single-point mutant strains based on the rDove and rBlackbird backbones (Fig. S4). Growth kinetics analysis revealed that substitutions F110L and G116R significantly accelerated viral replication during the first 24 hpi in the rDove background, followed by reduced replication at later time points. Reciprocal substitutions (L110F and R116G) similarly altered the growth characteristics of the rBlackbird strain (Fig. 4A and B).

Consistent with these findings, F110L and G116R substitutions increased the virulence of rDove, whereas L110F and R116G substitutions attenuated the virulence of rBlackbird, as reflected by changes in ICPI values (Table 2). In contrast, the remaining five HN substitutions produced minimal or no detectable effects on viral growth or ICPI (Table 2; Fig. 4A and B). Eight mutant viruses exhibiting altered ICPI values were selected for *in vivo* characterization (Table 2). In the rDove background, single F110L or G116R substitutions induced clinical signs and resulted in 30% and 40% mortality, respectively, and were associated with increased viral replication in multiple tissues (Fig. 4E through G; Table 3). In contrast, single L110F or R116G substitutions in the rBlackbird background did not alter mortality or tissue tropism, with all infected chickens succumbing to infection (Fig. 4H through J; Table 3).

To investigate whether combined substitutions could overcome this constraint, both mutations were introduced together into the Blackbird strain. In the rDove background, the double mutant rDove-HN_F110L/G116R_ reached titers approximately 10-fold higher than rDove at 12 hpi, followed by reduced replication between 36 and 60 hpi (Fig. 4C). In contrast, parental rBlackbird replicated to titers approximately 15-fold higher than rBlackbird-HN_L110F/R116G_ at 12 hpi, whereas the double mutant surpassed the parental virus at later time points (Fig. 4D). *In vivo*, the combined substitutions further increased virulence and tissue tropism in the rDove background relative to the single mutants, whereas the same substitutions significantly attenuated rBlackbird, reducing mortality to 40% and markedly decreasing viral titers, particularly in the brain (Fig. 4H through J; Tables 2 and 3).

To determine whether residues 110 and 116 exert similar effects in other NDV genotypes, a velogenic genotype VII strain, C22, was examined. The 116th amino acid of C22 HN is R (21). Introduction of a single L110F substitution into rC22 (rC22HN_L110F/G116_) significantly reduced the viral virulence (Table 2). Although the L110/R116 combination and F110/R116 combination attenuated the virus relative to rC22, they were more virulent than the F110/G116 combination.

These results demonstrate that HN residues F110 and G116 play a crucial role in reducing virulence and restricting tissue tropism in genotype IX NDV isolates from wild birds and further indicate that targeted modification of these residues can modulate virulence in other NDV genotypes.

### Amino acid residues 110 and 116 of the HN protein may influence virulence, potentially in a manner dependent on fusion activity

**Fig 5:**
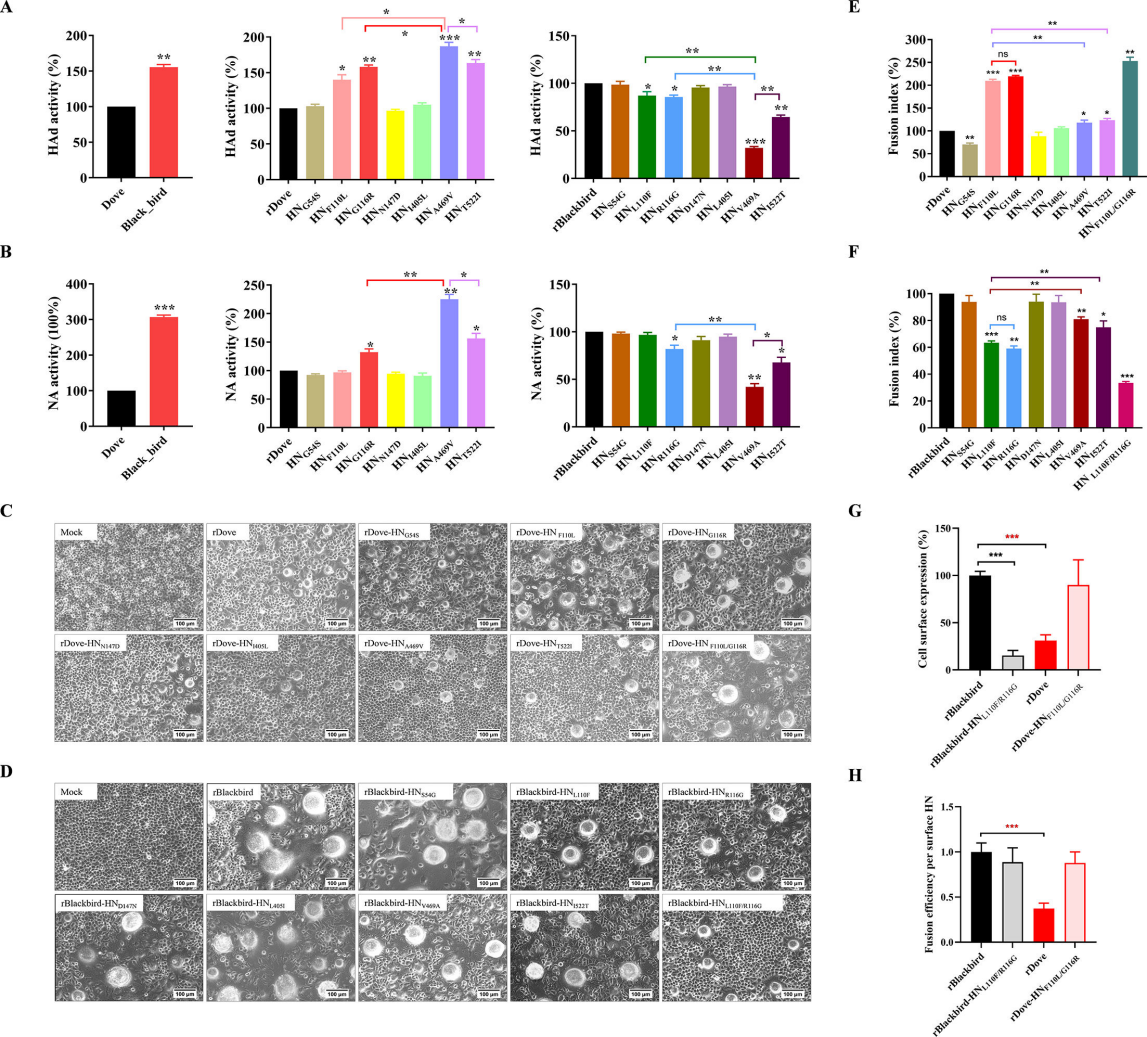
Amino acid residues 110 and 116 of the HN protein may influence virulence, potentially in a manner dependent on fusion activity. (**A**) NDV HAd Assay. At 24 hpi, 2% chicken blood was added to BHK-21 cells to test the hemadsorption activity of wild-type or mutant NDV. (**B**) NDV NA activity. At 8 hpi, BHK-21 cell lysates were incubated with a neuraminidase fluorescent substrate to measure NDV neuraminidase activity. (**C–F**) Fusion ability of original and mutant strains. At 24 hpi, syncytium formation in BHK-21 cells was imaged, and the average area of 40 syncytia was measured using ImageJ software. (**G**) The expression efficiency of the HN mutant proteins on the cell surface. (**H**) Fusion efficiency of original and mutant strains based on HN surface expression. Data are given as means ± standard deviation (SD) from three independent experiments. *P* values were calculated using Student’s *t*-test. An asterisk indicates a comparison with the indicated control. *P < 0.05; **P < 0.01; ***P < 0.001; ns: not significant.

To assess whether HN residues 110 and 116 influence NDV virulence through specific functional activities, hemagglutination (HAd), neuraminidase (NA), and membrane fusion assays were performed. The Blackbird strain exhibited higher HAd activity than the Dove strain (Fig. 5A). Mutations at residues 110 or 116 of the HN protein modulated the HAd ability of these two strains, although these effects were less pronounced than the changes observed with the 469th mutation (Fig. 5A). A similar trend was observed for NA activity, with the 469th mutation showing the most significant change among the seven single mutations (Fig. 5B).

In contrast, substitutions at residues 110 and 116 had a more pronounced effect on viral fusion activity. Specifically, F110L or G116R mutations in the Dove strain resulted in larger syncytia, whereas L110F or R116G mutations in the Blackbird strain led to smaller syncytia compared to wild-type or other mutant strains in infected cells (Fig. 5C and D). This effect was even more pronounced when both the 110th and 116th residues were mutated simultaneously. rDove-HN_F110L/G116R_ induced larger syncytia than rDove-HN_F110L_ or rDove-HN_G116R_, with a fusion index 2.5 times greater than that of the Dove strain (Fig. 5C and E). In contrast, rBlackbird-HN_L110F/R116G_ induced smaller syncytia in infected cells compared to rBlackbird-HN_L110F_ or rBlackbird-HN_R116G_, with a fusion index reduced to 33.5% of that observed in rBlackbird (Fig. 5D and F).

To explore whether these fusion phenotypes could be explained by differences in the HN abundance at the plasma membrane, HN cell-surface expression was measured for the parental and double mutant viruses. The HN surface expression level was significantly higher in rBlackbird than in rDove. Introduction of the F110L/G116R substitutions into the Dove background markedly increased HN surface expression to levels comparable to those of rBlackbird. In contrast, the reciprocal L110F/R116G substitutions in the Blackbird background significantly reduced HN surface expression to levels even lower than those of the parental rBlackbird strain (Fig. 5G). Notably, total cellular HN levels were comparable among these viruses (data not shown), suggesting that residues 110 and 116 may primarily affect the efficiency of HN accumulation at the cell surface rather than overall HN production.

To further examine whether the fusion phenotypes can be explained by differences in HN abundance at the plasma membrane, we calculated the fusion efficiency by dividing fusion activity by surface HN expression (Fig. 5H). Fusion efficiency for rBlackbird was normalized to 1, and values for the other strains were expressed relative to this reference. rBlackbird-HN_L110F/R116G_ showed fusion efficiency values close to 1, indicating that its reduced fusion activity is largely accounted for by decreased surface HN abundance. In contrast, rDove displayed a significantly lower fusion efficiency (~0.4). Given the low fusion ability of rDove and its reduced surface HN expression, this indicates that the decrease in fusion activity is more pronounced than the reduction in surface HN expression. Therefore, reduced surface HN abundance is a major contributor to the rDove fusion phenotype, but it may not fully account for the fusion defect, suggesting that additional determinants beyond surface expression may contribute. Interestingly, rDove-HN_F110L/G116R_ exhibited a normalized fusion efficiency close to 1, comparable to those of rBlackbird and rBlackbird-HN_L110F/R116G_. These results suggest that the increase in surface HN expression in the rDove-HN_F110L/G116R_ mutant significantly enhanced fusion activity, aligning with the observed improvement in fusion efficiency. This indicates that restoring surface HN expression in rDove via double mutations at residues 110 and 116 can recover fusion efficiency to levels comparable to those observed in rBlackbird.

Taken together, these results suggest that residues 110 and 116 are associated with differences in fusion phenotypes and that the observed changes in fusion largely correlate with changes in HN cell-surface expression. Therefore, the contribution of residues 110 and 116 to virulence-associated phenotypes is likely mediated, at least in part, by modulation of HN cell-surface abundance, with altered cell-cell fusion occurring secondary to these expression differences, rather than by major changes in HAd or NA activity.

### A modeled α-helix in the HN linker distinguishes Dove from Blackbird

**Fig 6:**
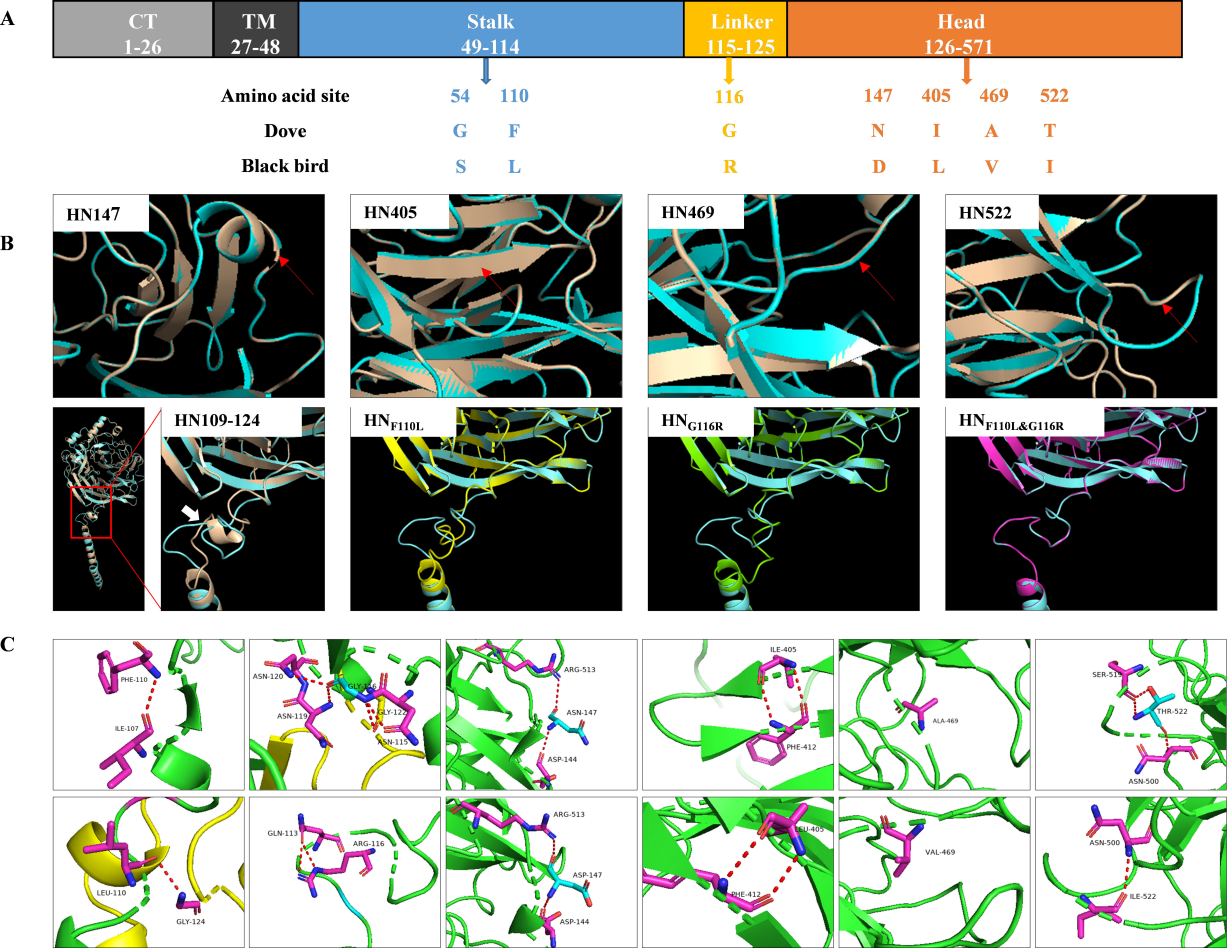
A modeled α-helix in the HN linker distinguishes Dove from Blackbird. (**A**) Diagram of the HN protein. The seven amino acid differences between the Blackbird and Dove strains are located in the stalk, linker, and head regions of the HN protein. (**B**) Structural comparison of the HN proteins from the Blackbird and Dove Strains. The structures of the HN proteins from both strains were modeled based on the structure of PIV5 HN (PDB: DABC) using SWISS-MODEL and compared with PyMOL. The colors blue, brown, yellow, green, and purple represent the wild-type Blackbird, wild-type Dove, F110L-mutated Dove, G116R-mutated Dove, and double-mutated Dove HN proteins, respectively. The red arrows point to the difference sites that show no significant structural changes. The red box highlights the linker region of the HN protein. The white arrows point to the newly formed α-helix structure. (**C**) Interaction of the 110th and 116th amino acids with other sites of the HN protein. The purple dashed line represents hydrogen bonds between amino acids.

To elucidate the molecular mechanism underlying the influence of double mutations at HN residues 110 and 116 on viral virulence, we analyzed the modeled HN structure. The differences in structure were localized to the region between residues 109 and 124 (Fig. 6A and B). In this region, the Dove strain HN (brown) exhibits an α-helix nestled between two random coils, whereas the Blackbird strain HN (cyan) presents a random coil in this region. Apart from this region, no significant structural differences were observed between the HN proteins of the two strains (Fig. 6B).

Given that this region includes the 110th and 116th residues, we further examined the structural changes following mutations in these positions. The 110th amino acid is located within the stalk domain, while the 116th amino acid resides in the linker region (Fig. 6A). The F110L (yellow) and G116R (green) mutations in the Dove strain led to a loss of the α-helix, transforming it into a random coil, although it still retained some structural differences compared to the Blackbird HN protein. However, when both mutations were combined (F110L/G116R, magenta), the conformation of the Dove HN protein fully matched that of the Blackbird HN protein. Hydrogen bonding analysis revealed that F110 in Dove forms a bond with I107 on the same chain, while L110 in Blackbird forms a bond with G124 from another subunit (Fig. 6C). Furthermore, G116 in Dove forms three hydrogen bonds with N115, N119, and N120 and one with G122 from another chain, whereas R116 in Blackbird forms a bond with Q113 on the same chain (Fig. 6C). These results suggest that differences at positions 110 and 116 affect the hydrogen bonding network and protein functionality.

Collectively, this result indicates that HN residues 110 and 116 in the Dove strain, by introducing an α-helix in the modeled head-stalk linker, may modulate HN flexibility and thereby account for the observed differences from Blackbird strain in fusion activity, tissue tropism, and pathogenicity.

### Chickens vaccinated with NDV Spotted Dove strain maintain high antibody titers for an extended period

**Fig 7:**
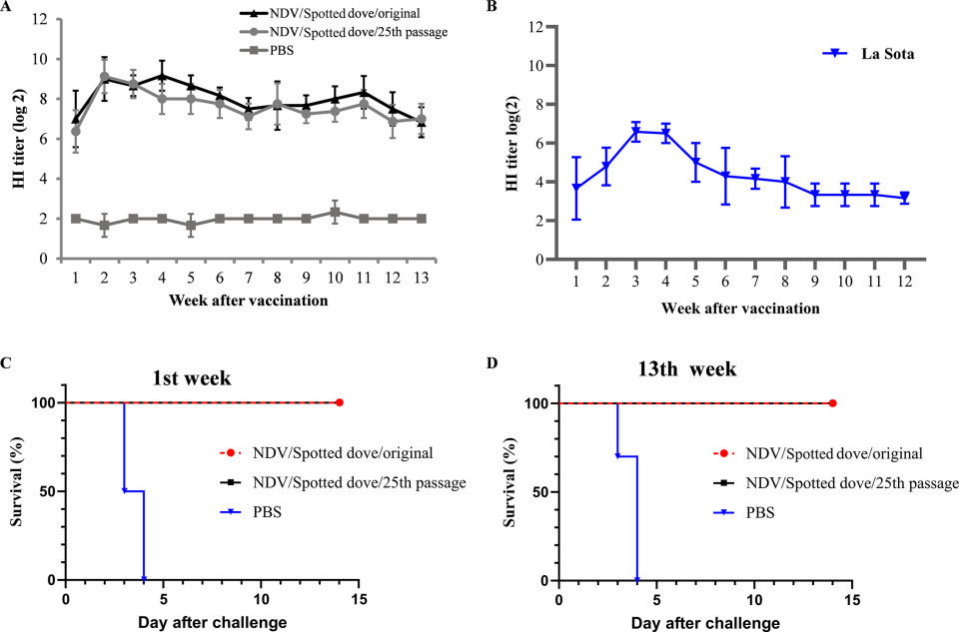
Chickens vaccinated with NDV Spotted Dove strain maintain high antibody titers for an extended period. (**A**) HI titer. Sera were collected weekly from chickens vaccinated with the original or passaged Dove strain, and NDV HI titers of three chicken were measured up to 13 weeks post-infection. (**B**) HI titer. Sera were collected weekly from chickens vaccinated with LaSota strain, and NDV HI titers of three chicken were measured up to 12 weeks post-infection. (**C and D**) Survival rate of chickens. Chickens vaccinated with the original or passaged Dove strain were challenged with a velogenic NDV F48E9 at the 1st week (**C**) and the 13th week (**D**) post-vaccination. The number of surviving chickens was recorded daily.

The Dove strain did not induce any clinical signs in chickens, prompting us to assess its potential as a vaccine candidate. To evaluate its pathogenicity, we passaged the strain through chicken embryos and monitored its stability. After 25 passages, the strain retained its lentogenic phenotype (data not shown). Subsequently, both the original and passaged strains were used to infect chickens, and hemagglutination inhibition (HI) titers were measured weekly to assess the immune response. The Dove strain induced HI titers of approximately 2^6^ 1 week post-vaccination, which increased to 2^9^ by the second week and remained stable between 2^7^ and 2^8^ through 13 weeks (Fig. 7A). To compare, chickens vaccinated with the LaSota strain exhibited HI titers of approximately 2^4^ 1 week post-vaccination, which peaked at around 2^7^ by the third and fourth weeks, and gradually declined to 2^3^ by week 12 (Fig. 7B). At both the 1st and 13th weeks post-vaccination, chickens immunized with Dove were challenged with the velogenic F48E9 strain. Clinical signs and mortality were monitored daily for 14 days following challenge, and no clinical symptoms or deaths were observed in the Dove strain-vaccinated group (Fig. 7C and D).

These results indicate that the Dove strain exhibits robust immunogenicity, providing rapid and long-lasting protection against velogenic NDV in vaccinated chickens.

## DISCUSSION

In this study, we investigated two genotype IX NDV isolates from wild birds that are nearly identical at the genome level yet show divergent pathogenicity in chickens. Reverse-genetics mapping identifies the HN protein, specifically residues 110 (stalk) and 116 (linker), as key modulators of fusion, tissue distribution, and virulence. Modeling suggests that the dove-derived HN forms an α-helix within the head-stalk linker that is absent in the blackbird HN, and functional assays show backbone-dependent, bidirectional effects on replication and virulence despite a velogenic-type F-cleavage motif.

Genotype IX NDV exhibits a distinctive evolutionary profile characterized by early emergence, restricted geographic distribution, and limited genetic diversification (22). Phylogenetic analyses indicate that early genotype IX viruses circulated primarily in poultry, with wild bird-derived strains emerging only recently and sharing >99.5% nucleotide identity with the earliest poultry isolate, consistent with minimal adaptive divergence following host transitions (Fig. 1A). BEAST-based relaxed clock molecular evolutionary analysis further revealed a markedly lower substitution rate for genotype IX NDV than for globally circulating genotypes such as genotype VII (Fig. 1A) (22–27). This unusually slow evolution, together with the absence of sustained international spread and the frequent occurrence of asymptomatic infections in wild birds, argues against long-term endemic circulation in avian wildlife (20, 25–33).

While genotype IX NDV strains are isolated from asymptomatic wild birds, most of these isolates exhibit significant pathogenicity in chickens (6–8, 22). Notably, certain strains, such as the Blackbird isolated from the Eurasian blackbird, induced 100% mortality in infected chickens. In contrast, the Dove strain, isolated from the spotted dove, displayed markedly reduced virulence, failing to induce clinical symptoms in 3-week-old chickens (Fig. 2). A comparative amino acid analysis of all viral proteins between the lentogenic Dove strain and the velogenic Blackbird isolate revealed only 12 divergent residues, which were localized to P, HN, and L protein (Table 1). Notably, the Dove strain retains the canonical virulent F protein cleavage motif (¹¹²RRQRRF¹¹⁷), which is widely regarded as a primary molecular determinant of NDV virulence (14), yet it exhibited attenuation, suggesting that factors beyond the F protein contribute to the strain’s unique tissue tropism and pathogenicity. Given that the observed amino acid differences were restricted to the P, HN, and L proteins, we next sought to determine which of these proteins was responsible for the divergent virulence and tissue tropism observed between the Dove and Blackbird strains. Exchange of the HN gene alone was sufficient to largely change the tissue tropism and virulence in both viral backgrounds, whereas substitution of the P and L genes produced only minor or negligible effects (Fig. 3). These findings indicate that HN plays a dominant role in regulating systemic spread and disease severity in genotype IX NDV. Subsequent mutagenesis studies identified residues 110 and 116 in the HN protein as critical determinants of the attenuated tissue tropism and reduced pathogenicity of the Dove strain. The observed effects were most pronounced in terms of tissue tropism, with the rDove-HN_F110L/G116R_ strain exhibiting expanded viral replication across multiple organs, while rBlackbird-HN_L110F/R116G_ showed a marked reduction in viral titers, particularly in the brain (Fig. 4). These findings indicate that the presence of specific amino acids in HN influences the virus’s ability to infect different tissues.

Our results highlight the critical role of HN residues 110 and 116 in modulating NDV virulence, primarily through their impact on surface HN expression and fusion activity (Fig. 5). rBlackbird exhibited larger syncytia and high surface HN expression. However, when mutations at residues 110 and 116 were introduced, resulting in rBlackbird-HN_L110F/R116G_, syncytia significantly shrank, and surface HN expression decreased. Similarly, rDove displayed smaller syncytia and lower surface HN expression. When the F110L/G116R mutations were introduced in rDove, the resulting mutant virus exhibited syncytia comparable in size to those of rBlackbird and a marked increase in surface HN expression (Fig. 5C through G). These findings are consistent with the strong correlation between surface HN expression and fusion activity, as evidenced by the changes in syncytium size observed in rBlackbird, rBlackbird-HN_L110F/R116G_, and rDove-HN_F110L/G116R_. The size of syncytia in these strains is primarily determined by the level of surface HN expression (Fig. 5H). However, in the case of rDove, the reduction in fusion activity appears to be more pronounced than the decrease in surface HN expression, suggesting that additional factors beyond its surface HN accumulation may contribute to the observed phenotypic changes. Given that HN availability at the cell surface is a key determinant of productive fusion and subsequent cell-to-cell spread, variation at residues 110 and 116 may contribute to virulence-associated phenotypes by altering HN surface expression and the resulting fusion capacity. This highlights the central role of HN in regulating the fusion process, which is crucial for viral spread within the host.

Notably, in the rDove background, single mutations at HN residues 110 and 116 both markedly increased mortality and tissue viral load. In contrast, in the rBlackbird background, single mutations at these positions resulted in 100% mortality in chickens and failed to reduce viral titers in tissues (Fig. 4F, G, I and J). Structural studies have defined the overall architecture of paramyxovirus HN proteins, including the multifunctional globular head and the stalk domain, and for NDV specifically, the HN ectodomain contains a four-helix bundle stalk (34, 35). In this structural context, computational modeling suggests that residues 110 and 116 form an α-helix within the head-stalk linker of the Dove strain, a feature absent in the Blackbird strain (Fig. 6B). This model provides a possible explanation for why, in the Blackbird background, single mutations at positions 110 and 116 cannot restore the α-helix that is unique to the Dove strain, with the helix only being formed in the case of the double mutation. In the model, loss of this helical element is associated with changes in local hydrogen-bonding patterns and may influence conformational preferences within the linker region (Fig. 6B and C). Consistent with this computational model, the double mutations that break the helix in rDove (i) tripled HN surface density, (ii) enlarged syncytia 2.5-fold, and (iii) amplified replication in organs, meanwhile the reciprocal swaps which recover the helix in rBlackbird reduce fusion index and viral load. Thus, the residues 110 and 116 modulate the efficiency of HN accumulation at the plasma membrane, thereby shaping fusion activity and *in vivo* spread. This model aligns with evidence that fusion triggering by paramyxovirus attachment proteins is modular and can be governed by stalk-associated determinants and that receptor-induced triggering can proceed through stepwise spatiotemporal transitions (36, 37). Accordingly, increasing the availability of HN at the cell surface could raise the likelihood of productive triggering events and thereby enhance cell-cell spread, tissue dissemination, and virulence.

An analysis of the HN protein amino acid sequences from 1,488 NDV strains revealed that only eight strains contain both the F110 and G116 residues. When these two residues were introduced simultaneously into the HN protein of the genotype VII virulent strain C22, the resulting virus exhibited significantly attenuated pathogenicity in chickens (Table 2). This observation raises the question of whether the co-existence of F110 and G116 is inherently unstable, which could explain their rare combination in natural NDV isolates. To address this, the Dove strain was serially passaged through chicken embryos for 25 generations. Sequencing of the passaged virus demonstrated stable maintenance of both F110 and G116, despite the emergence of other mutations in the HN protein (data not shown). Pathogenicity assays confirmed that the passaged virus retained its attenuated phenotype, suggesting that the critical virulence-determining residues, F110 and G116, remain co-stabilized within the genetic context of the Dove strain.

Although current Newcastle disease vaccines provide satisfactory immunoprotective efficacy, they are limited by factors such as the need for approximately 3 weeks post-vaccination to induce adequate antibody levels, short-lived high-titer antibody persistence, and the requirement for multiple doses (38–41). Both the original Dove strain and its 25th-generation chicken embryo-passaged variant can induce protective antibodies (HI titers of approximately 2^7^) in 3-week-old chickens within 7 days post-infection (Fig. 7A). These antibodies remain at effective levels for up to 13 weeks (HI titers ≥ 2^6^), providing 100% protection against challenges with virulent strains during this period (Fig. 7). Compared with vaccine strain LaSota, chickens exhibited HI titers of approximately 2^4^ 1 week post-vaccination, which peaked at around 2^7^ by the third and fourth weeks, and gradually declined to 2^3^ by week 12 (Fig. 7B). Importantly, this attenuation mechanism appears stable and biologically meaningful. The Dove strain maintained low pathogenicity after extensive embryo passage while eliciting robust and durable protective immunity in chickens, outperforming the widely used LaSota vaccine in sustaining antibody titers.

Taken together, our findings show that two genotype IX NDV isolates from wild birds exhibit strikingly different virulence in chickens despite near-identical genomes and identify HN residues 110 and 116 as key determinants of attenuation. The effects of these residues are strongly linked to changes in HN cell-surface abundance and downstream fusion activity, tissue dissemination, and pathogenicity. Computational modeling further suggests that the F110/G116 combination may be associated with a local α-helical element in the HN head-stalk linker, providing a plausible structural context for these phenotypes. Collectively, these results support further evaluation of the attenuated dove-derived virus as a candidate for Newcastle disease vaccine development and provide a rationale for exploring HN-linked attenuation mechanisms in the design of NDV-based viral vectors, pending additional studies of safety, genetic stability, and efficacy.

## MATERIALS AND METHODS

### Animals

Three-week-old specific-pathogen-free (SPF) chickens and 9- to 11-day-old SPF embryonated chicken eggs were purchased from JINAN SPAFAS POULTRY CO., LTD (Jinan, China). Animal experiments were reviewed and approved by the Ethics Committee at Northwest A&F University. All experiments with live virus were conducted in biosafety level 2 (BSL-2) facilities with appropriate personal protective equipment and containment protocols, in compliance with institutional and national regulations for handling select agents.

### Cells and viruses

The chicken embryo fibroblast cell line (DF-1) and baby hamster kidney cell line (BHK-21) were obtained from the American Type Culture Collection (ATCC, Manassas, VA). Both cell lines were cultured in Dulbecco’s minimal essential medium (DMEM; Thermo, Waltham, USA) supplemented with 10% fetal bovine serum (FBS; Gibco, USA), 100 U/mL penicillin, and 0.1 mg/mL streptomycin and grown in a 37°C incubator supplied with 5% CO2. Both NDV/Dove strain and NDV/Blackbird strain were stored in our laboratory and propagated in the allantoic cavity of 9- to 11-day-old SPF embryonated chicken eggs. Virus titers (50% tissue culture infectious doses/mL, TCID_50_/mL) were calculated using the Reed and Muench method.

### ICPI test

The pathogenicity of the chimeric viruses was determined by ICPI test in 1-day-old chicks following standard procedures; fresh allantoic fluid was diluted 10 times, and then 0.05 mL of each virus per chick was inoculated into ten 1-day-old SPF chicks via the intracerebral route. The birds were observed for clinical signs and mortality once every 24 h for 8 days. At every observation, they were scored 0 if normal, 1 if sick, and 2 if dead. The ICPI value was the mean score per bird per observation. The values of virulent (velogenic) viruses approach 2 and avirulent (lentogenic) viruses give values close to 0.

### Pathogenicity in 3-week-old SPF chickens, virus titration, and histopathology

Furthermore, the pathogenicity of the NDVs was evaluated in 3-week-old SPF chickens by the eye and nasal drops route. Briefly, groups of 13 3-week-old SPF chickens were inoculated with 0.2 mL containing 10^6^ TCID_50_ of virus or phosphate-buffered saline (PBS) via the oculonasal route. The birds were observed and scored daily for clinical symptoms of disease (0 for normal, 1 for sick, 2 for paralysis/twitching/wing drop, 3 for prostration, and 4 for death) until 14 dpi. Three birds from each group were euthanized at 4 dpi, and turbinal bone, lung, trachea, spleen, brain, proventriculus, duodenum, cecal tonsil, and bursa of Fabricius were collected for virus titration and histopathology examination. For virus titration, 0.1 g of the tissue samples was homogenized in PBS containing antibiotics, and virus titers (TCID_50_/g) were calculated on BHK-21 cells. For histopathology examination, tissue samples (lung, brain, duodenum, and bursa of Fabricius) were fixed in 4% paraformaldehyde. The fixed tissues were sectioned and stained with hematoxylin-eosin. The remaining 10 chickens in each group were given daily clinical scores, which were calculated as mentioned before (18). Survival was monitored and recorded daily until 14 dpi. A mean score per virus group per day was generated for comparisons.

### Immunization and challenge studies in SPF chickens

The 21-day-old SPF chickens were randomly divided into four groups. Group 1 was inoculated with PBS as a control (*n* = 23). Group 2 received the original Dove strain (*n* = 23), Group 3 was inoculated with the 25th-passage Dove strain (*n* = 23), and Group 4 was vaccinated with LaSota (*n*=3). Each chicken received a virus dose of 10^6^ 50% egg infective dose (EID_50_). Ten chickens from Groups 1, 2, and 3 were selected for NDV challenge experiments at both 1 week (*n* = 10) and 13 weeks (*n* = 10) post-vaccination and were inoculated with 10^6^ TCID_50_ of the F48E9 strain. Survival was monitored and recorded daily for 14 dpi. The remaining chickens from each group were not challenged, and serum was collected weekly for antibody testing by HI. Each bird received 200 µL liquids via the oculonasal route.

### Viral genome sequencing and phylogenetic analysis

Viral genomic RNA was extracted from the infectious allantoic fluid using the EasyPure Viral DNA/RNA Kit (*TRANS*, Beijing, China) according to the manufacturer’s instructions. NDV genome sequencing was carried out by Genscript Biotechnology Co. (Nanjing, China). Alignment and comparison of the nucleotides and amino acid sequences between them were carried out using Clustal W in the Lasergene sequence analysis software package (MegAlign). The Markov Chain Monte Carlo (MCMC) method was employed to reconstruct the maximum clade credibility (MCC) tree and estimate the mutation rate using BEAST 1.10.4. A Bayesian phylogenetic analysis was conducted with the Relaxed Clock model. F gene sequences were analyzed through MCMC to calculate the mutation rate, with a total length of 3 × 10^8^ steps and sampling every 1 × 10^3^ steps. Convergence of all parameters (effective sample size [ESS] >200, burn-in of 10%) was assessed using Tracer v1.7.1. The final MCC tree was generated with TreeAnnotator and visualized using Figtree v1.4.4.

### Plasmid construction, transfection, and virus rescue

The construction of the full-length antigenomic cDNA clone of NDV Dove dne Blackbird strain is shown in Fig. S1B, and seven fragments were generated by reverse transcription-PCR (RT-PCR) of RNA that had been purified from allantoic fluid from infected eggs and cloned in pBR322 vector through eight restriction enzyme (RE) sites, AscI, PmeI, PacI, AsiSI, NotI, SnaBI, SacII, and RsrII. The leader region was preceded by a T7 RNA polymerase promoter that added three G residues to the 5′ end of the antigenome, and the trailer region was flanked by a self-cleaving hepatitis delta virus (HDV) antigenome ribozyme, followed by a T7 RNA polymerase terminator (18). All restriction sites are created, except SacII. In addition, the naturally occurring RsrII sites in the HN and L ORFs were eliminated without amino acid changes. The cDNA was completely sequenced, and the constructed plasmid was designated pBR-rDove and pBR-rBlack_bird. The P, HN, and L genes were substituted through the restriction enzyme sites noted above. And the site mutations in the HN gene were conducted by overlap PCR. These recombinant viruses were recovered in BHK-21 cells. In brief, the full-length cDNAs, a helper plasmid pCMV-NPPL simultaneously expressing the NP, P, and L proteins, and a plasmid pCACCS-T7 as a source of T7 polymerase were co-transfected into BHK-21 cells. Four days after transfection, the cell culture supernatants were harvested and injected into the allantoic cavity of 9-day-old SPF embryonated chicken eggs, and then the viruses were confirmed by hemagglutination (HA) assay. The positive allantoic fluid was then passaged five times in 9-day-old SPF embryonated chicken eggs, and total RNA was isolated from the fifth passage. The rescued viruses were confirmed by sequencing.

### Virus growth kinetics

The growth kinetics of NDVs were determined on DF-1 cells under multiple-cycle growth conditions. The cells were infected with NDVs at a multiplicity of infection (MOI) of 0.01. And the supernatant was collected at 12 h intervals and replaced by equal volumes of DMEM containing 1% FBS until 60 h post-infection (hpi). Virus titers (TCID_50_/mL) were determined as described above.

### Fusion index assay

The fusogenic abilities of viruses were evaluated by the fusion index assay described by Yan et al (42). Briefly, viruses were inoculated onto confluent BHK-21 cells in 6-well plates at an MOI of 0.01. After 1 h adsorption at 37°C under 5% CO2, cells were washed with PBS, and 2 mL of DMEM containing 1% methylcellulose was added into the culture. At 24 hpi, 40 syncytia were randomly photographed using an inverted microscope (IX73, OLYMPUS, Tokyo, Japan) at 200-fold magnification, and the areas of the syncytia were counted by Image J software. The fusion index was calculated as the mean fusion regions of 40 syncytia and was normalized to the parent virus, rDove or rBlack_bird, set as 100%.

### Hemadsorption (HAd) assay

The HAd assay was performed as previously described to determine the receptor-binding activity of the HN protein (43). Briefly, viruses were inoculated onto the monolayers of BHK-21 cells at an MOI of 1. At 8 hpi, cells were washed gently with cold PBS and incubated with 2% chicken red blood cell (CRBC) in PBS at 4°C for 1 h. Subsequently, the cells were washed thoroughly to remove unbound RBCs, and 100 μL of 50 mM NH4Cl solution was added to lyse the CRBCs that bound to the infected cells. The lysate was then centrifugated at 12000 rpm for 5 min at 4°C, and the absorbance of the supernatant was measured at a wavelength of 540 nm. The values were expressed relative to rDove or rBlack_bird, set as 100%.

### Neuraminidase (NA) assay

For NA assay, viruses were inoculated at an MOI of 1 onto confluent BHK-21 cells. At 8 hpi, the cells were detached from the plates using 50 mM EDTA-PBS and centrifuged at 3,000 rpm for 5 min at 4°C. Subsequently, 20 μL of radioimmunoprecipitation assay (RIPA) buffer (Solarbio, Beijing, China) was added to the cells, and then the samples were freeze-thawed three times at −80°C, followed by centrifugation at 12000 rpm for 5 min at 4°C. Then, the remaining steps were performed according to the manufacturer’s instructions for the neuraminidase assay kit (Beyotime, Shanghai, China). Samples collected from virus-free cells served as controls. The NA values were normalized relative to rDove or rBlack_bird, set as 100%.

### Flow cytometry assay

BHK-21 cells were infected at 0.01 MOI with wild-type or double mutant viruses. After 24 h, the medium was removed, and the monolayer was detached with 0.25% trypsin-EDTA (Gibco, Grand Island, NE). Digestion was stopped with 10% FBS-DMEM, and the cells were pelleted (1,500 rpm, 5 min), washed once with PBS, and resuspended in 200 µL of 4% BSA for 30 min at RT to block. Then, 100 µL of primary anti-HN monoclonal antibody (2G8) (1:100 in 4% BSA), produced in our lab (44), was added and incubated for 2 h at room temperature (RT) with gentle mixing every 10 min. Cells were washed twice and stained with 100 µL of Goat Anti-Mouse IgG H&L (Alexa Fluor 488) (Abcam, Shanghai, China) (1:500 in 4% BSA) for 1 h in the dark, again with periodic mixing. After two final washes, cells were fixed in 500 µL PBS containing 1% paraformaldehyde for 10 min, passed through the nitrocellulose filter membrane into a flow tube, and the cell surface fluorescence intensity was detected by a flow cytometer (BD Biosciences, San Jose, CA).

### Structure analysis of NDV HN protein

The three-dimensional structures of NDV HN proteins were predicted by SWISS MODEL based on the PIV5 HN protein (pdb: DABC). Comparisons and annotations of structures were performed with PyMOL (The PyMOL Molecular Graphics System, Version 1.7.4 Schrödinger, LLC).

### Statistical analysis

Statistical analysis was conducted using GraphPad Prism 7 software (GraphPad Software, Inc., CA, USA). The unpaired *t*-test and one-way ANOVA were used to evaluate the significance of the differences between experimental groups. *P* values ≤ 0.05 were considered statistically significant.

## Data Availability

All data supporting the findings of this study are available within the article. Further details can be obtained from the corresponding author.

## SUPPLEMENTAL MATERIAL

Fig. S1 jvi.00071-26-s0001.tif Amino acid diversity between NDV isolated from Blackbird and Dove strains.

Fig. S2 jvi.00071-26-s0002.tif Rescue of the Blackbird and Dove strains.

Fig. S3 jvi.00071-26-s0003.tif Fusion ability of the chimeric virus strains.

Fig. S4 jvi.00071-26-s0004.tif Confirmation of mutated strains.

Supplemental legends jvi.00071-26-s0005.pdf Descriptive legends for Fig. S1 to S4.

Table S1 jvi.00071-26-s0006.pdf Isolation and pathogenicity of genotype IX NDV strains.

Table S2 jvi.00071-26-s0007.pdf Isolation of genotype VII NDV strains.

ASM does not own the copyrights to Supplemental Material that may be linked to, or accessed through, an article. The authors have granted ASM a non-exclusive, world-wide license to publish the Supplemental Material files. Please contact the corresponding author directly for reuse.
