# Supplementary figures and images for: Genotype IX Newcastle disease virus isolated from wild birds is attenuated by hemagglutinin-neuraminidase mutation
Source: J Virol. 2026 May 20;100(6):e00071-26. doi: 10.1128/jvi.00071-26 (PMC13288932; doi:10.1128/jvi.00071-26)

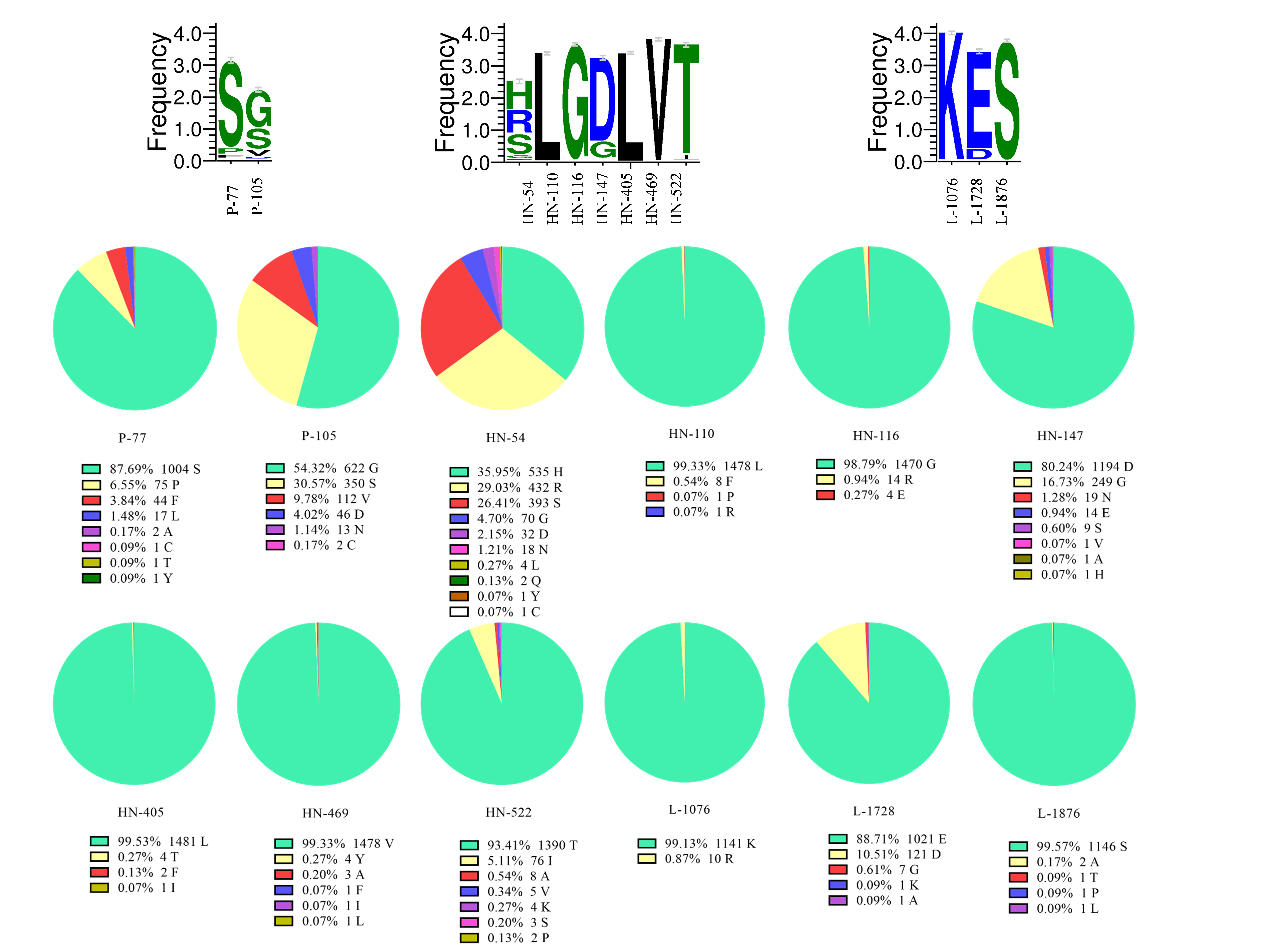

Supplement: Fig. S1 — Amino acid diversity between NDV isolated from Blackbird and Dove strains. [file jvi.00071-26-s0001.tif]

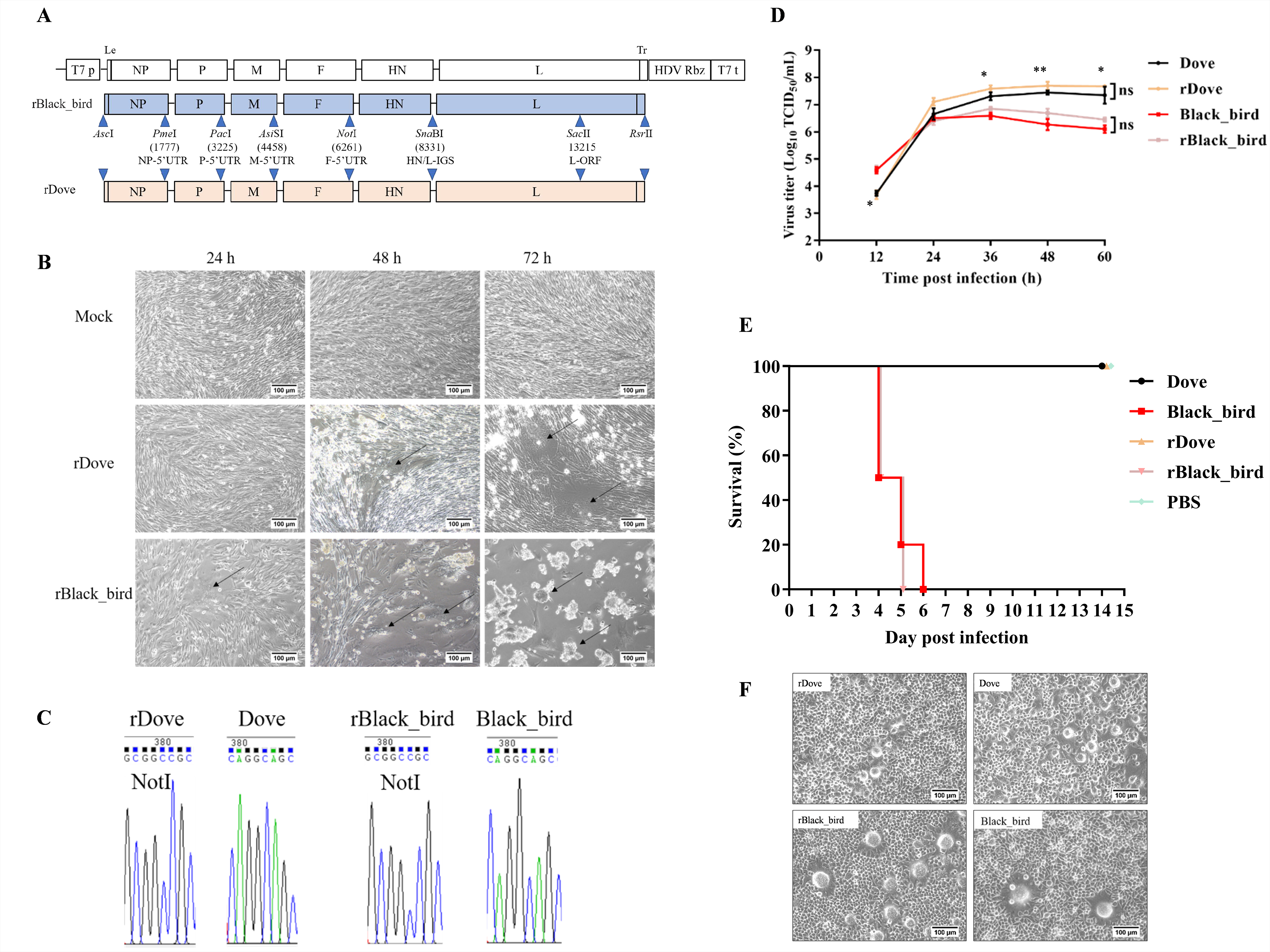

Supplement: Fig. S2 — Rescue of the Blackbird and Dove strains. [file jvi.00071-26-s0002.tif]

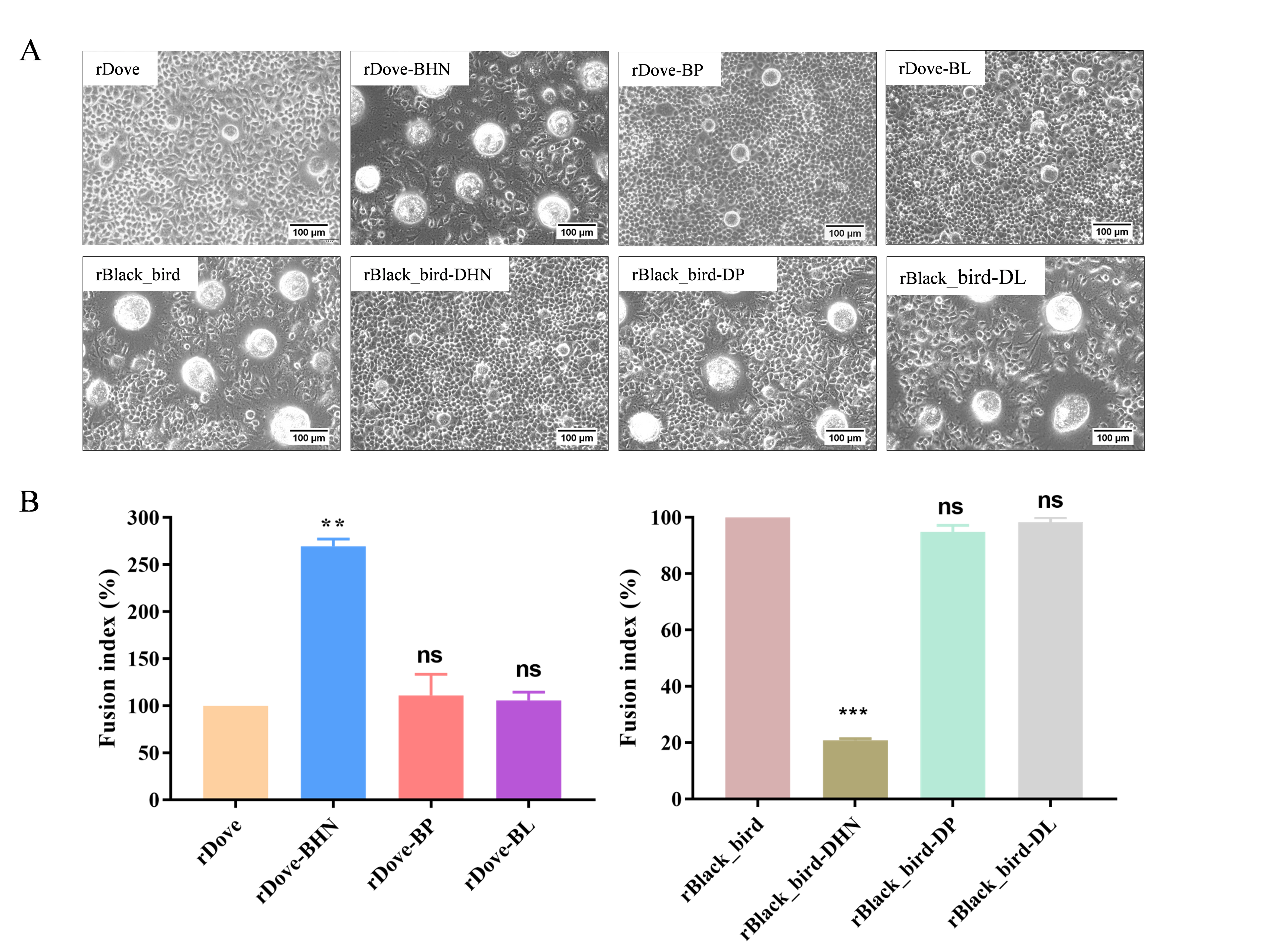

Supplement: Fig. S3 — Fusion ability of the chimeric virus strains. [file jvi.00071-26-s0003.tif]

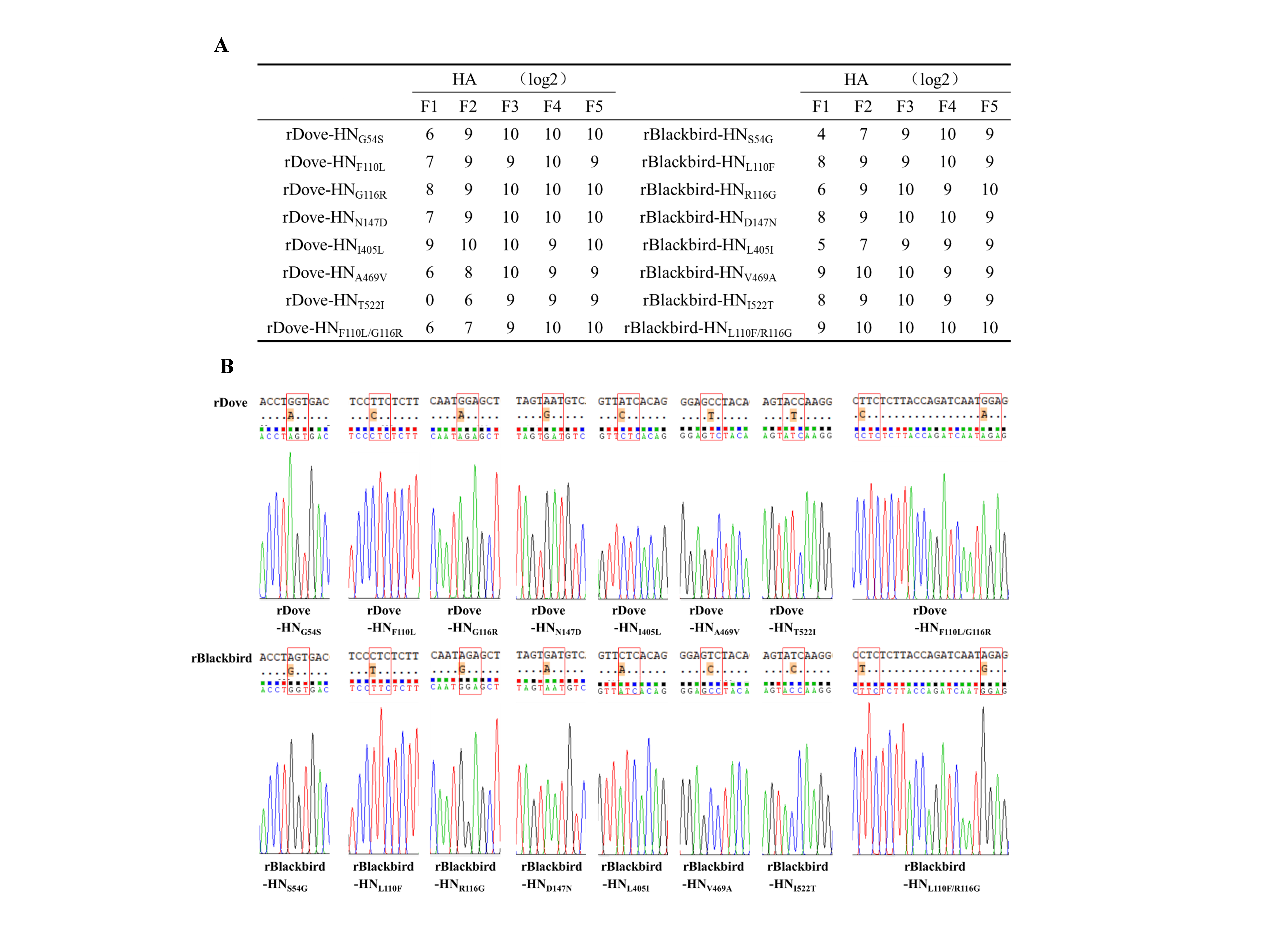

Supplement: Fig. S4 — Confirmation of mutated strains. [file jvi.00071-26-s0004.tif]
