# Supplementary material for: Genotype IX Newcastle disease virus isolated from wild birds is attenuated by hemagglutinin-neuraminidase mutation
Source: J Virol. 2026 May 20;100(6):e00071-26. doi: 10.1128/jvi.00071-26 (PMC13288932; doi:10.1128/jvi.00071-26)
Supplement: Supplemental legends — Descriptive legends for Fig. S1 to S4. [file jvi.00071-26-s0005.pdf]

FIG S1. Amino acid diversity between NDV isolated from Eurasian Blackbird (Blackbird Strain) and Spotted Dove (Dove Strain). A total of 12 amino acid differences were identified across all proteins, including 2 in the P protein, 7 in the HN protein, and 3 in the L protein between the two strains. The diversity of these amino acids was analyzed by comparing the P, HN, and L proteins of 1145, 1488, and 1151 NDV strains, respectively.

FIG S2. Rescue of the Blackbird and Dove strain. (A) Schematic diagram of plasmids containing Full-Length cDNA of the Blackbird or Dove Strain. Restriction enzyme sites were incorporated into the genome for plasmid construction. (B) Viral rescue. Full-length cDNA and helper plasmids were transfected into BHK-21 cells, and CPE were observed every 24 h. Arrows indicate syncytia formation. (C) Confirmation of rescued Virus. The rescued virus was sequenced and identified by the presence of the introduced *Not* I restriction enzyme site. (D) Viral replication *in vitro*. The replication capacity of the wild type or chimeric strains was evaluated in DF-1 cells. (E) Survival rate of infected chickens. The daily mortality rate was recorded for 3-week-old chickens infected with wild type or chimeric strains. (F) Syncytium formation by virus. BHK-21 cells were infected with wild type or chimeric strains to induce syncytium formation. Data are given as means ± standard deviation (SD) from three independent experiments. P values were calculated using Student's t-test. An asterisk indicates a comparison with the indicated control. *$P < 0.05$; **$P < 0.01$; * **$P < 0.001$; ns: not significant.

FIG S3. Fusion ability of the chimeric virus strains. (A) Syncytium formation by virus. BHK-21 cells were infected with chimeric strains to induce syncytium formation. (B) The average area of 40 syncytia was measured and compared between the original and chimeric strains. P values were calculated using Student's t-test. An asterisk indicates a comparison with the indicated control. *$P < 0.05$; **$P < 0.01$; * **$P < 0.001$; ns: not significant.

FIG S4. Confirmation of mutated strains. (A) Viral passage in chicken embryos. After rescue, the mutated strains were passaged in chicken embryos, and HA titers were determined. (B) Sequencing of mutated strains. After five passages, the mutated strains were sequenced to confirm the presence of the expected mutation.
