# Supplementary material for: Genotype IX Newcastle disease virus isolated from wild birds is attenuated by hemagglutinin-neuraminidase mutation
Source: J Virol. 2026 May 20;100(6):e00071-26. doi: 10.1128/jvi.00071-26 (PMC13288932; doi:10.1128/jvi.00071-26)
Supplement: Table S1 — Isolation and pathogenicity of genotype IX NDV strains. [file jvi.00071-26-s0006.pdf]

TABLE S1 Isolation and pathogenicity of Genotype IX NDV strains

NDV Isolate	GenBank Accession No.	Date	Host	Pathotype
F48E9	MG456905	1946	Chicken	Velogenic
FJ/1/85/Ch	FJ436304	1985	Chicken	Velogenic
ZJ/1/86/Ch	FJ436303	1986	Chicken	Velogenic
JS/1/97/Ch	FJ436305	1997	Chicken	Velogenic
CK/FS/SS/N/1997	MF278924	1997	Chicken	-[#]
JS/1/02/Du	FJ436306	2002	Duck	Velogenic
XBT14	JX677561	2002	Duck	Velogenic
China/NDV03-45-641/2003	JQ697741	2003	-	-
Poultry/China/04-23/C12/647/2004	MH392220	2004	-	-
Duck/CH/GD/FS-06	KC750153	2006	Duck	-
SpottedDove/China/08	KC934170	2008	Spotted dove*	Lentogenic
Blackbird/China/08	KC934169	2008	Eurasian blackbird*	Velogenic
Whooper swan/China/08	KC424428	2008	Whooper swan*	Mesogenic
Peafowl/China/08	KC424427	2008	Peafowl*	Mesogenic
White-cheeked starling/China/08	KC424431	2008	White-cheeked starling*	Mesogenic
GD09-2	HQ317394	2009	Duck	Velogenic
Duck/CH/GD/NH/10	KF219497	2010	Duck	Velogenic
Layer/China/Yulin/10	KP027403	2010	Chicken	Velogenic
Layer/China/Chang'an/10	KP027404	2010	Chicken	Velogenic
JS0920	JN698890	2011	Duck	-
Duck/China/Guangxi19/2011	KC920893	2011	Duck	Velogenic
Common moorhen/Ch/GD/GZ201/2014	KY788668	2014	Common moorhen*	Velogenic
Spotted dove/Ch/GD/GZ232/2014	KY788669	2014	Spotted dove*	Velogenic
Duck/Guangdong/YF18/2014	KT381605	2014	Duck	Velogenic
Spot-billed duck/Ch/GD/GZ12/2014	KY788666	2014	Spot-billed duck*	Velogenic
GD14	MF581293	2014	Goose	-
MDK/FS/SS/485/2014	MF278931	2014	Duck	-
Black_swan/Guangdong/W76/2019	MT668583	2019	Black swan*	-
NDV-BJ	KF915807	-	-	Velogenic
HBNU/LSRC/F3	KC246549	-	-	Lentogenic
LuoY	AY341061	-	-	-
TJ03	DQ227244	-	-	-
JS06	DQ858356	-	-	-
BY	EF589136	-	Chicken	Velogenic
FW	EF589135	-	Chicken	Velogenic

* Healthy wild bird

[#] No information
