# Supplementary material for: Genotype IX Newcastle disease virus isolated from wild birds is attenuated by hemagglutinin-neuraminidase mutation
Source: J Virol. 2026 May 20;100(6):e00071-26. doi: 10.1128/jvi.00071-26 (PMC13288932; doi:10.1128/jvi.00071-26)
Supplement: Table S2 — Isolation of genotype VII NDV strains. [file jvi.00071-26-s0007.pdf]

TABLE S2 Isolation of Genotype VII NDV strains

Accession	Collection date (Year)	Country	Genotype
JN986837	1993	Netherlands	VII
KJ782375	1997	China	VII
JN618348	1997	China	VII
JN599167	1999	China	VII
AB853927	1999	Japan	VII
FJ754273	2000	China	VII
MK124761	2000	China	VII
JX193075	2002	China	VII
FJ872531	2002	China	VII
KU295454	2003	Ukraine	VII
DQ485230	2003	China	VII
KR074407	2004	Malaysia	VII
JN986838	2004	South Africa	VII
KM977903	2005	China	VII
JN618349	2005	China	VII
KY776598	2006	China	VII
KC542895	2006	China	VII
MH377283	2007	Israel	VII
KC542896	2007	China	VII
KP189357	2008	Russia	VII
MK340930	2008	China	VII
KC542905	2009	China	VII
JN400895	2009	China	VII
KJ607170	2010	China	VII
HQ697255	2010	Indonesia	VII
KY076032	2011	Pakistan	VII
KC542908	2011	China	VII
KT760568	2012	China	VII
JX532092	2012	Pakistan	VII
KU295455	2013	Ukraine	VII
KM670337	2013	Pakistan	VII
MH105251	2014	China	VII
KP776462	2014	Pakistan	VII
MF437287	2015	Pakistan	VII
KU845252	2015	Pakistan	VII
MG717686	2016	Egypt	VII
KX765879	2016	China	VII
MG871466	2017	Iran	VII
MK342603	2017	China	VII
MH614933	2018	Jordan	VII
MH432252	2018	Belgium	VII
